# Multi-Omics Approach for Studying Tears in Treatment-Naïve Glaucoma Patients

**DOI:** 10.3390/ijms20164029

**Published:** 2019-08-18

**Authors:** Claudia Rossi, Ilaria Cicalini, Maria Concetta Cufaro, Luca Agnifili, Leonardo Mastropasqua, Paola Lanuti, Marco Marchisio, Vincenzo De Laurenzi, Piero Del Boccio, Damiana Pieragostino

**Affiliations:** 1 Center for Advanced Studies and Technology (CAST), University ‘‘G. d’Annunzio’’ of Chieti-Pescara, 66100 Chieti, Italy; 2 Department of Medical, Oral and Biotechnological Sciences, University ‘‘G. d’Annunzio’’ of Chieti-Pescara, 66100 Chieti, Italy; 3 Department of Medicine and Aging Science, “G. d’Annunzio” University of Chieti-Pescara, 66100 Chieti, Italy; 4 Department of Pharmacy, University ‘‘G. d’Annunzio’’ of Chieti-Pescara, 66100 Chieti, Italy; 5 Opthalmic Clinic, Ss Annunziata Hospital, 66100 Chieti, Italy

**Keywords:** proteomics, extracellular vesicles, metabolomics, lipidomics, tears, biomarkers, amino acids, acylcarnitines, glaucoma

## Abstract

Primary open-angle glaucoma (POAG) represents the leading cause of irreversible blindness worldwide and is a multifactorial, chronic neurodegenerative disease characterized by retinal ganglion cell and visual field loss. There are many factors that are associated with the risk of developing POAG, with increased intraocular pressure being one of the most prevalent. Due to the asymptomatic nature of the disease, the diagnosis of POAG often occurs too late, which necessitates development of new effective screening strategies for early diagnosis of the disease. However, this task still remains unfulfilled. In order to provide further insights into the pathophysiology of POAG, we applied a targeted metabolomics strategy based on a high-throughput screening method for the determination of tear amino acids, free carnitine, acylcarnitines, succinylacetone, nucleosides, and lysophospholipids in naïve to therapy glaucomatous patients and normal controls. Also, we conducted proteomic analyses of the whole lacrimal fluid and purified extracellular vesicles obtained from POAG patients and healthy subjects. This multi-omics approach allowed us to conclude that POAG patients had lower levels of certain tear amino acids and lysophospholipids compared with controls. These targeted analyses also highlighted the low amount of acetylcarnitine (C2) in POAG patient which correlated well with proteomics data. Moreover, POAG tear proteins seemed to derive from extracellular vesicles, which carried a specific pro-inflammatory protein cargo.

## 1. Introduction

Primary open angle glaucoma (POAG) is a group of chronic multifactorial neurodegenerative diseases of the optic nerve characterized by progressive loss of retinal ganglion cells (RGC) and visual field (VF) impairment. The increased intra-ocular pressure (IOP) is the most important risk factor for the development and progression of the RGC loss [1].

To date, POAG represents the leading cause of irreversible blindness [2]. The global prevalence of POAG for population aged 40–80 years is 3.05%. Worldwide, the number of affected subjects is estimated to dramatically increase in the next decades [3]. Given these estimates and the rapid increase in ageing populations, POAG has a high public health and economic burden. These facts support the importance and the need for screening strategies and early POAG diagnosis.

The diagnosis of POAG requires the presence of typical optic disc signs and consistent VF alterations. Unfortunately, the disease is asymptomatic in nature and the VF loss is not evident until the RGC decreases to at least 30%. Thus, the diagnosis of POAG often occurs late leading to a high need of strategies and biomarkers for an early-stage diagnosis.

Due to the easy and non-invasive sampling process, tears have progressively gained a huge interest in the last decades since they may be considered as a reservoir of potential biomarkers of different ocular diseases, including POAG [4,5].

Given the location, which is quite close to the trabecular meshwork (the site where the initial damage occurs leading to IOP increase), tears may potentially contain several biomarkers of POAG. In more detail, POAG-related biomarkers may directly come from the aqueous humor, which soaks the trabecular meshwork and the anterior chamber of the eye, after trans-scleral percolation [6].

As known, proteomics and metabolomics approaches of biological fluids represent an useful tool for biomarker discovery in clinical applications. In this context, the use of lacrimal fluid in multi-omics investigation has been recently described in ocular disorders and in other pathological conditions [4,7,8,9]. Moreover, the growing application of mass spectrometry, as targeted metabolomics strategy in routine clinical laboratories, provides an opportunity to quantify a panel of analytes in a single measurement process. Indeed, the major high-throughput clinical applications of mass spectrometry are: Neonatal screening for inborn errors of metabolism by the determination of specific metabolites and second-tier tests for the diagnostic confirmation of several metabolic diseases [10,11,12,13]. In addition, proteomics investigation results in further insights on the pathological mechanisms and the involved pathways through the study of tear proteome profile. The aim of the present study was to analyze, with a multi-omics approach, the protein and metabolic content of the whole tear film and from sorted extracellular vesicles (EVs) in a small clinical cohort of newly diagnosed naïve to therapy glaucomatous patients and of healthy subjects, in order to highlight alterations of lacrimal molecular composition possibly related to the pathological condition.

## 2. Results

### 2.1. Demographic and Clinical Data

Table 1 lists demographic and clinical data of enrolled patients and controls (CTRLs). No significant differences were found between glaucomatous patients and CTRLs for all demographic parameters; conversely, IOP mean resulted significantly different between groups (*p* < 0.01).

### 2.2. Targeted Metabolomics by Direct Infusion Mass Spectrometry Analysis

**Figure 1:**
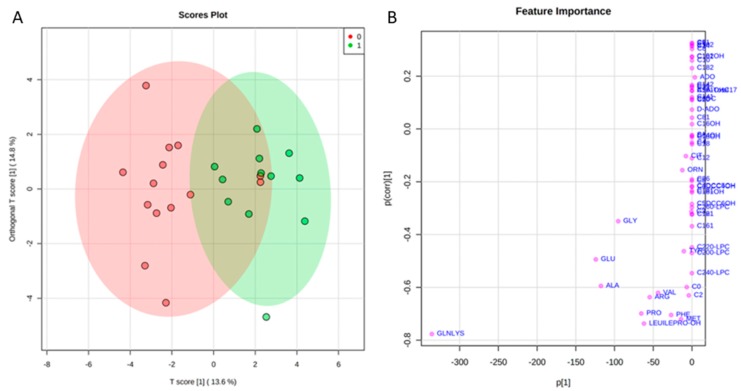
(**A**) shows the bidimensional scores plot obtaining by orthogonal partial least squares - discriminant analysis (orthoPLS-DA) statistical model. The red and green portion of the graph indicates the 95% confidence region for the separation between the clinical groups in consideration: POAG patients (red dots) and CTRL (green dots). The plot in Panel (**B**) visualizes the variables influence in an orthogonal PLS-DA model, combining the covariance and correlation loading profiles. At the bottom center the most discriminating analytes in the construction of the orthogonal PLS-DA model are shown.

**Figure 2:**
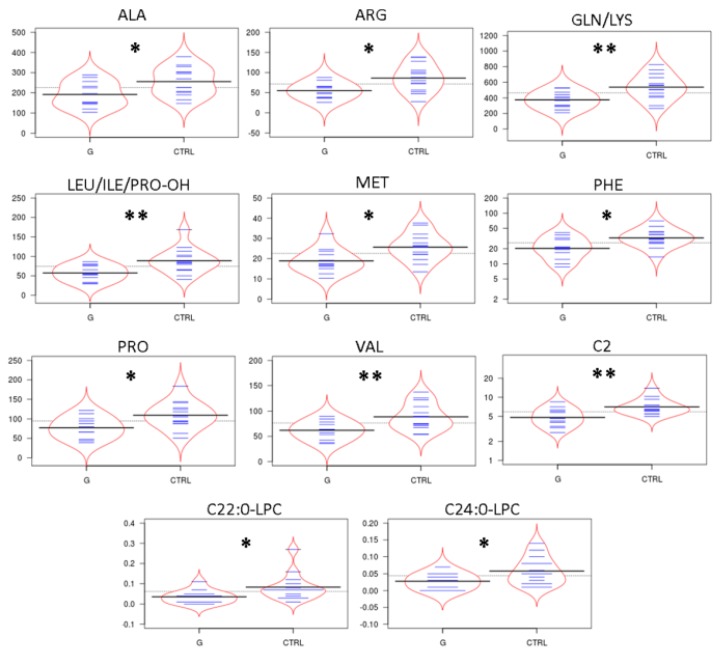
In Figure 2 we report the distribution of the significantly different amino acids (AAs), acylcarnitines (ACCs) and lysophosphatidylcholine (LPCs) in the comparison between tear samples from glaucoma patients (G) versus tear samples from CTRL, visualized as the beanplots. In these plots, the polygon shape (in red line) represents the density trace computed using a log-transformation of each variable, and inside to that, a scatter plot shows all individual measurements (blue lines). * means *p* < 0.05, ** means *p* < 0.01 at the Student’s t-test and/or Mann Whitney *U*-test.

Levels of tear amino acids (AAs), acylcarnitines (ACCs), C0, succinylacetone, nucleosides, and lysophospholipids in naïve to therapy POAG patients and CTRL were determined by direct infusion mass spectrometry (DIMS) analysis and underwent a multivariate statistical approach. In particular, orthogonal partial least squares—discriminant analysis (orthoPLS-DA) was built using the entire metabolic dataset, by Metaboanalyst 3.0 software. As reported in Figure 1 panel A, the bidimensional scores plot shows a clear separation between the clinical groups: POAG patients (red dots) and CTRLs (green dots). The metabolites that best drive the separation between tears of POAG patients and CTRLs are depicted in Figure 1 Panel B. The plot visualizes the variables influence in an orthoPLS-DA model, also indicating, at the bottom center of the plot, the most discriminating analytes in the separation between the study groups. In order to define data distribution in each group, the data matrix was statistically processed performing D’Agostino and Pearson omnibus normality test. Once normality was accepted, Student’s t-test was performed, alternatively the Mann Whitney test was carried out to assess the significantly expressed variables between the groups. Except for acetylcarnitine (C2) and C22:0 lysophosphatidylcholine (C22:0-LPC) for which non-normal distribution was observed and therefore Mann Whitney test was applied, the Student’s t-test was used for all other metabolites with gaussian distribution. Thus, the discriminant tear metabolites are represented in the bean-plots in Figure 2. In particular, we found that the POAG patient group (G) shows consistently lower levels of some tear AAs as alanine (Ala), arginine (Arg), glutamine/lysine (Gln/Lys), leucine/isoleucine/hydroxyproline (Leu/Ile/Pro-OH), methionine (Met), phenylalanine (Phe), proline (Pro), valine (Val), of acetylcarnitine (C2), and of two lysophospholipids as C22:0-LPC and C24:0 lysophosphatidylcholine (C24:0-LPC) compared with CTRLs. In contrast, we did not find significant differences in the other tear ACCs and nucleosides between the study groups. Furthermore, tear tyrosine (Tyr) and free carnitine (C0) showed a trend towards lower levels in the patient group compared with CTRLs, as summarized in Supplementary Table S1. In addition, we observed no significant differences in metabolomic data between males and females in POAG patients (Table S2).

### 2.3. Tear Proteomics

Pooled tears samples from a selected and a well classified cohort of patients affected by POAG and CTRLs were used for proteomic analysis as described in the Methods section. In Table S3 the identified proteins in both pathophysiological conditions (123 proteins for CTRL patients and 103 proteins for POAG tears) are reported. The list of identified proteins in at least two replicates for each condition (reported in Table S3 in Supplementary Files) was subjected to functional enrichment protein analysis through the FunRich Database tool.

**Figure 3:**
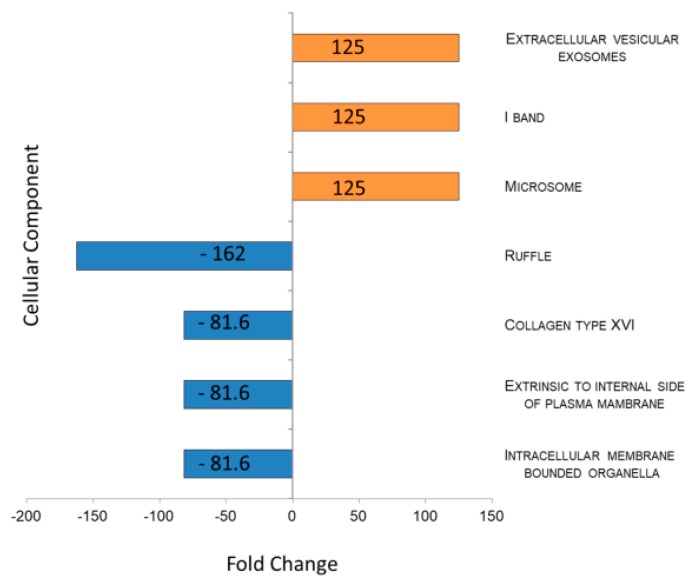
Histogram representing enrichment analysis performed on Cellular Component using FunRich database. Enriched and Depleted Cellular component in tears of POAG patients are reported in blue and in orange, respectively. Gene Ontology code for each CC reported in figure: EV and Exosomes: GO:0070062; I BAND: GO:0031674; MICROSOME: GO:0005792; RUFFLE: GO:0032587; COLLAGEN TYPE XVI: GO:0005597; EXTRINSIC TO INTERNAL SIDE OF PLASMA MEMBRANE: GO:0031234; INTRACELLULAR MEMBRANE-BOUNDED ORGANELLE: GO:0043231.

Figure 3 shows the results, sorted by fold change, of the comparison between cellular component (CC) classification obtained through FunRich investigation in POAG patients as compared to CTRLs. Data showed an inhibition of “intracellular membrane bounded organella” CC in POAG and the “extracellular vesicles exosomes” CC 125 times more enriched in POAG in respect to CTRL biofluids (*p*-value = 0.004). Contrariwise, the CC called RUFFLE (GO:0032587), portion of the plasma membrane surrounding a ruffle, resulted depleted in POAG tears (*p*-value = 0.007).

These results suggest that POAG biofluids probably collect a high number of functional EVs. Following this result, total and intact EVs in POAG and CTRL tears were identified as LCD+/Phalloidin- and sorted for proteomics characterization, as previously reported [14].

### 2.4. Tear EVs Proteomics

**Figure 4:**
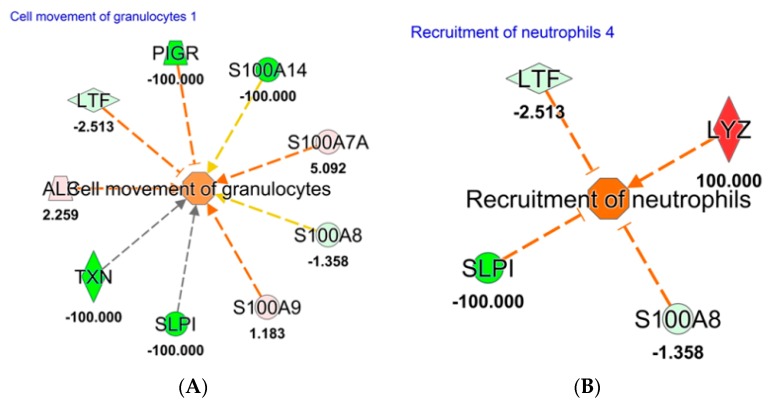
Downstream analysis, using Ingenuity Pathway software, based on proteins identified and quantified in EVs sorted in POAG and CTRL tears. Red and green color indicates genes significantly increased and decreased in expression, respectively, while the number represent the fold change log. The dotted lines represent indirect relationships. Symbols used in the figure represent: Vertical rhombus, enzyme; trapezoid, transporter; circle, other; concentric circles, complex/group. (**A**): Cell movement of Granulocytes; (**B**): Recruitment of Neutrophils. Abbreviation: Polymeric immunoglobulin receptor (*PIGR*); Antileukoproteinase (*SLPI*); Thioredoxin (*TXN*); Albumin (*ALB*); Lactotransferrin (*LTF*); and Lysozyme C (*LYZ*).

To study the influence of protein cargo derived from EVs, total and intact EVs were identified as LCD+/Phalloidin− and separated by instrumental cell sorting from POAG and CTRL tears, as already described [14]. Therefore, 1.0 × 10^6^ sorted EVs from each condition have been analyzed by a shotgun proteomics approach in triplicate. The list of identified proteins in at least two replicates for each condition is reported in Table S4. Quantitative proteomics data were obtained through MaxQuant software. Raw data were compared by Ingenuity Pathways analysis (IPA software), uploading proteins fold change log (POAG versus CTRL) for Core Analysis. Activated downstream regulators in POAG tears were depicted in Figure 4, showing how protein cargo of EVs in POAG subjects is able to trigger “inflammatory response” (*p*-value = 1.22 × 10^−11^). Such biofunction is realized through an hyper-activation of “cell movement of granulocytes” (*p*-value = 9.03 × 10^−7^, z score = 1.02) and of “recruitment of neutrophils” (*p*-value = 1.34 × 10^−3^, z score = 1.89), as reported in Figure 4 Panel A and B, respectively.

**Figure 5:**
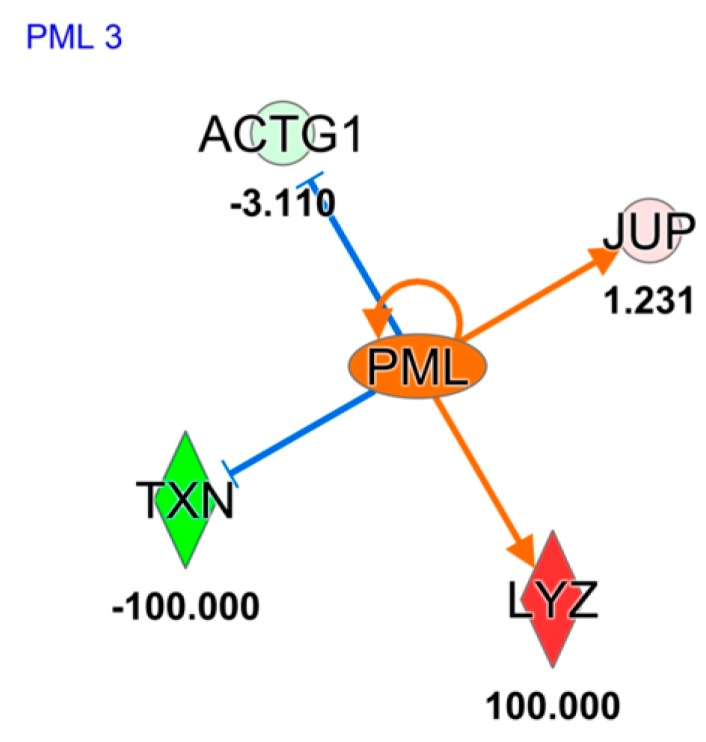
Upstream regulator analysis, based on proteins identified and quantified in EVs sorted in POAG and CTRL tears, using Ingenuity Pathway software. Orange and blue shapes represent predicted activation or inhibition, respectively. The predicted relationship between genes may lead to direct activation (orange solid lines) or direct inhibition (blue solid lines). Red and green color indicates genes significantly increased and decreased in expression, respectively, while the number represent the fold change log. Symbols used in the figure represent: Enzyme symbolized as vertical rhombus and “other” protein symbolized as circle. Abbreviations: Junction plakoglobin *(JUP*); Thioredoxin (*TXN*); Actin, cytoplasmic 2 (*ACTG1*); and Lysozyme C (*LYZ*).

Differential proteins obtained from EVs analysis of both conditions, were also used for “upstream regulator analysis” by IPA. Such investigation, principally based on the input dataset, identifies known regulators and generates direct or indirect relationships between genes. In Figure 5 the activation of protein PML (*PML*) gene, (z score = 1.97) in POAG EVs patients, is reported. Such activation is mainly driven by junction plakoglobin (*JUP*), Thioredoxin (*TXN*), Actin, cytoplasmic 2 (*ACTG1*), and Lysozyme C (*LYZ*). *PML* is responsible for cysteine-type endopeptidase activity involved in the apoptotic process.

## 3. Discussion

POAG describes a group of chronic and multifactorial optic neurodegenerative disorders characterized by progressive loss of RGC and VF. The major risk factors are ocular conditions such as elevated IOP, myopia, pseudo-exfoliation, thin central cornea, and systemic factors such as cardiovascular diseases, diabetes, and ageing (the risk increases after 40 years). From a pathophysiological point of view, the mechanical stress induced by the increased IOP and the vascular impairment leads to the RGC loss and the consequent VF alterations [15].

In this context, multi-omics approaches may define molecular profiles describing the disease phenotype through the integration of the analysis of small-molecules, like metabolites, and of functional molecules, as proteins, with statistical models. Such a strategy is a promising tool for biomarker discovery, applicable to asymptomatic diseases such as POAG. Considering the global incidence of POAG and the great impact of an early stage disease diagnosis on the patient quality of life, screening strategies for early diagnosis and treatment of POAG are highly required [16]. To date, only few studies described the metabolomic profiles in POAG using mass spectrometry [15,17,18]. Many of these studies have been performed on the blood of individuals with POAG. In particular, some studies compared the plasma metabolome of individuals with POAG using an untargeted metabolomics approach [19] or using a standardized targeted metabolomic approach [15]. Some other studies reported the metabolomics of the aqueous humor in a rat model of POAG using nuclear magnetic resonance [20]. The last investigation described metabolomic profiling of aqueous humor in POAG patients by a standardized targeted metabolomics strategy [18]. Thus, to our knowledge, the present study, is the first that investigates the metabolic profiling of tears in naïve to therapy glaucomatous patients, even if only a small cohort of tear samples were analyzed.

The tear film is a thin layer of aqueous, lipid, and mucin fluid between the eyelids. Being directly associated with the corneal surface, the tear film is an accessible source in studying ocular surface disorders. Moreover, tears collection is a non-invasive procedure that facilitates large-scale and translational clinical studies. More recently “omics” technologies were used to identify molecular alterations in tears associated to different disorders not always eye-related [9]. Indeed, the global profiling of metabolites from this bio-fluid is of particular interest as “omics” approach to provide an overview of the dynamic biochemical processes that occur in POAG.

Previous studies reported a significant increase of some AAs in plasma and in aqueous humor of POAG patients. In particular, Leruez et al. described an increase of plasma Tyr, Met, and Arg, and Buisset et al. reported increased levels of Gln, Glycine, Ala, Leu, Ile, Pro-OH, and Acetyl-Ornithine in POAG patients aqueous humor [15,18]. In contrast, our study revealed consistently lower levels of some tear AAs as Ala, Arg, Gln/Lys, Leu/Ile/Pro-OH, Met, Phe, Pro, and Val in the POAG patient group compared with CTRLs. Moreover, also tear Tyr showed a trend toward lower levels in the patient group compared with CTRLs. In line with our findings, Vohra et al. also described reduced serum levels of AAs in patients with normal-tension POAG compared to healthy subjects and suggested them as potential markers for normal-tension POAG [21]. In particular, Vohra et al. evaluated alterations in serum AA levels in POAG patients compared with CTRLs to further understand a possible link between fluctuating oxygen availability, energy metabolism and glaucomatous neurodegeneration. Since AAs, as energy substrates, are crucial for ATP production, they supposed that energy substrates may be limited in patients with POAG and that the cellular mechanisms to either take up or metabolize AAs may be dysfunctional in POAG patients and related to the characteristic glaucomatous neurodegeneration [21]. Thus, the observed metabolic alterations may be of great interest in serving as potential diagnostic markers, paving the way to novel targets to treat and prevent glaucomatous neurodegeneration. Furthermore, in agreement with Calandrella et al., we found lower levels of C2 and a trend toward lower levels of C0 in tears of POAG patients compared with CTRLs. As reported by Calandrella et al., L-carnitine and its acetylated derivative (C2) have a neuroprotective, antiapoptotic and antioxidant actions, being able to improve the mitochondrial metabolism [22]. In fact, they explored the possibility that L-carnitine may modulate the effects of ocular hypertension. Thus, treatment with L-carnitine may protect against disease-related stress.

Interestingly, it has been reported that alterations in lysophospholipid biology might be linked to altered IOP in POAG [23,24], and, only very recently, the distribution profile of different phospholipids, ceramides and glycosphingolipids was investigated both in normal and glaucomatous eyes through mass spectrometry [25,26]. These studies showed a decrease of the levels of both phospholipids and glycosphingolipids in the glaucomatous aqueous humor and trabecular meshwork, thus indicating their potential role in the pathophysiology of POAG. Intriguingly, our targeted metabolomics approach by DIMS analysis also revealed lower levels of two lysophospholipids as C22:0-LPC and C24:0-LPC in the lacrimal fluid of POAG patients compared to CTRLs. All these data suggest that a comprehensive lipidomics approach of tears might provide significant insights into their role in POAG pathobiology and elevated IOP.

Considering that ageing is a major risk factor of POAG, Leruez et al. interestingly tempted to speculate that the glaucoma-related metabolic alterations may lead to a biochemical phenotype of premature ageing [15].

To complete the functional characterization of POAG tears, we studied proteins through proteomics approach. Lacrimal protein functional classification highlighted as “extracellular vesicles exosomes” is 125 times more active in POAG as compared to CTRL biofluids, allowing the hypothesis that EVs studies may improve the sensitivity of tear biomarker discovery in POAG. Therefore, we applied our recent published method [14] for sorting tear EVs, with the aim of studying their protein cargo. Quantitative proteomics data, studied through IPA software, highlighted as identified proteins in EVs are able to trigger the “inflammatory response” through a significant increase of recruitment of neutrophils. The “recruitment of neutrophil” is mainly driven by overexpression of *LYZ*, already reported in our previous work on whole tear fluid from POAG patients naïve to therapy [27]. Here, we prove as this high amount of *LYZ* in patients is strongly due to EVs amount. This result may be integrated with the low amount of C2 highlighted in targeted metabolomics approach, which is able to attenuate neuroinflammation, as already demonstrated [28]. Therefore, we may speculate as low levels of C2, together with high amount of *LYZ* in EVs may be involved in inflammation machinery activated in POAG. Our data are consistent with a recent study which highlighted reduced concentrations of taurine in POAG patients. Actually, taurine has an important cytoprotective function against oxidative stress, inflammation, and osmoprotection, and its concentration decreases with age, in particular in the cornea and lens [18].

Moreover, proteomics data suggest that tears EVs seem to be released by cells with a hyper-activation of *PML*, as showed by upstream analysis in Figure 5. Indeed, *PML* involvement in POAG etiology was already described by Nakano et al., who performed a genome-wide association study using 201 exfoliation POAG and 697 CTRL, identifying 34 genome-wide significant single-nucleotide polymorphisms distributing in lysyl oxidase-like 1 gene, TBC1D21, and *PML* at the 15q24.1 locus [29]. In addition, it has been reported that murine *PML*-null primary cells are resistant to *TGF-β*-induced apoptosis and that cytoplasmic *PML* is an essential activator of transforming growth factor-β (*TGF-β*) signaling [30]. This result is particularly relevant, considering that many recent studies have suggested that TGF-β plays a major role in the POAG process [31]. Considering that *TGF-β* is widely involved in trabecular meshwork sclerosis and in fibrotic phase of POAG [32,33,34], and that it has already been proposed as a potential therapeutic target [31], the description of such *PML-TGF- β* axis may help in highlighting new putative pharmacological targets for POAG treatment.

Since POAG clinical signs only appear when the disorder is in advanced stage, a fast screening test on an easily accessible biofluid, such as tears or serum, would be helpful for diagnosis and follow up of glaucoma progression. Thus, the correlation of POAG biomarkers and disease progression would be a really interesting aspect to be investigated in future studies. Although we recognize that the number of participants included in the study is limited, we think that these data shed light on the role of integrated omics approaches in the study of POAG, and on the potentiality of studying EVs in such biological fluid.

## 4. Material and Methods

### 4.1. Patient Enrollment

16 patients with a recent diagnose of POAG were consecutively included in the study, from November 2017 to March 2019, at the Ophthalmology clinic of the University G. d’Annunzio of Chieti-Pescara, Italy.

Inclusion criteria for POAG were the followings: Best corrected visual acuity (BCVA) ≥ 8/10, a refractive error ≤ 3 D, mean untreated IOP ranging higher than 22 mmHg, central corneal thickness (CCT) ranging from 520 to 570 μm, full-threshold VF test showing at least three contiguous points on the total deviation probability plot at the < 2 % level, with POAG Hemifield Test results outside normal limits in at least one eye, and signs of glaucomatous optic neuropathy consistent with VF alterations. Exclusion criteria were: History of ocular surface, eyelids, or intra-ocular inflammatory diseases during the last 6 months; history of topical or systemic therapy in the last 3 months that could interact with the ocular surface status; previous ocular surgery or laser trabeculoplasty, trauma, advanced stage POAG, contact lens wear, and pregnancy.

17 healthy subjects served as CTRL and had to respect the followings inclusion criteria: Normal ocular surface and eyelids, a normal anterior and posterior segment at the macroscopic slit-lamp evaluation, BCVA ≥ 8/10, refractive error ≤3 D, and mean IOP < 18mmHg with a CCT ranging from 520 to 570 µm. Exclusion criteria were: History of topical or systemic therapy potentially affecting the ocular surface and eyelids, ocular or systemic diseases in the last 3 months, any diseases potentially affecting the eyelids, ocular surgery, pregnancy, and contact lens wear.

The clinical and demographic features of the involved patients in the study are listed in Table 1.

All patients were informed about the procedures and provided written informed consent to participate in the study. In order to protect human subject identity a number code was employed for specimen identification. The present study was conducted according to Declaration of Helsinki (64^TH^ WMA General Assembly, Fortaleza, Brazil, October 2013).

### 4.2. Samples Collection

All tear samples were collected at the *Ophthalmology clinic of the University G. d’Annunzio of Chieti-Pescara, Italy.* Tear samples were collected on graduated Schirmer’s strips as we previously described [7,9]. The Schirmer strips were purchased from EasyOpht (Busto Arsizio, VA, Italy). Briefly, tears were collected on the graduated paper strips pulling the lower lid gently downward for 5 minutes. Following, the strip was placed in a 2.0 mL Eppendorf tube and stored at −80°C.

### 4.3. Tear Sample Extraction for Targeted Metabolomics Investigation

The determination of 14 AAs, 35 ACCs, C0, succinylacetone, 2 nucleosides, and 4 lysophospholipids was performed in tear samples by the addition of isotopically labelled internal standards (ISs) for each analyte of interest prior to the extraction, according to the principle of isotope dilution internal standardization.

Schirmer’s strips imbibed by tears were cut and transferred into microcentrifuge tube (Eppendorf®, Hamburg, Germany). After adding the extraction solution (300 µL) containing ISs, each tear samples were shaken in a thermo mixer (750 rpm, 45 °C, 30 min) [35,36].

The internal standards, as well as the extraction solution, were obtained from the NeoBase 2 Non-derivatized MSMS Kit (Perkin Elmer Life and Analytical Sciences, Turku, Finland). 100 μL for each extracted sample was transferred to a vial and leave at room temperature for an hour for succinylacetone derivatization [37].

### 4.4. Metabolites Analysis by Direct Infusion Mass Spectrometry (DIMS)

The DIMS analysis for the evaluation of metabolite profile in tear strip samples was performed using an Ultra Performance Liquid Chromatography Tandem Quadrupole Mass Spectrometry (UPLC/MS/MS) system (Acquity UPLC I-Class coupled to a Xevo TQD, Waters Corp., Manchester, UK). Mass spectra were acquired in positive electrospray ionization, using multiple reaction monitoring (MRM) as acquisition mode. Data are processed by using MassLynx V4.1 and NeoLynx Software (Waters Corp.). 10 μL were injected into the ion source and the run time was 1.1 minutes, injection-to-injection. The specific mass spectrometer setting conditions are reported in our previous works [37,38]. A detailed list of analyzed metabolites, their ionization source settings, and their abbreviations as used in the text are available in Supplementary Table S5. The mass spectrometer ionization source settings were optimized for maximum ion yields for each analyte.

### 4.5. Tear Sample Extraction for Proteomics Investigation

Tear Schirmer’s strip samples were cut into 2–3 mm paper pieces and transferred into a 0.5 mL microcentrifuge tube (Eppendorf®, Hamburg, Germany), paying attention to wash the required equipment with EtOH before and after each sample preparation. After adding 200 µL of 0.01M PBS, Phosphate Buffered Saline (Sigma-Aldrich, St. Louis, MI, USA), each sample was gently mixed (22 °C, 10 min, 700 rpm) in a Thermomixer (Eppendorf®, Hamburg, Germany). After, the 0.5 mL tubes containing the piece of the strip in PBS were cut off on the bottom, put into another 2.0 mL microcentrifuge tube (Eppendorf®, Hamburg, Germany) and centrifuged (room temperature, 5 min, 13000 rpm). The extracted liquid (195 µL) was transferred into 1.5 mL microcentrifuge tube (Eppendorf®, Hamburg, Germany), aliquoted and used for subsequent FC analyses in order to sort pure EVs or for proteomics investigation.

Proteomics analyses were executed on whole lacrimal fluid from 5 subjects belonging to the two different clinical groups. Tear proteins were first extracted, pooled and then quantified by Bradford assay (Bio-Rad, Hercules, CA, USA), using Bovine Serum Albumin (BSA, Sigma-Aldrich, St. Louis, MI, USA) standard for the calibration curve. 50 µg of proteins was digested for each clinical group.

### 4.6. EV Separation by Instrumental Cell Sorting

EVs were separated (100 µm nozzle) from whole samples on the basis of their positivity pattern to Lipophilic Cationic Dye (LCD), Custom Kit (Cat. 626267, BD Biosciences), their negativity to phalloidin as previously reported by using a FACSAria III cell sorter (BD Biosciences, Franklin Lakes, NJ, USA) [14]. 1 × 10^6^ purified EVs from POAG and CTRL tears were analyzed by proteomics.

### 4.7. Label Free Proteomics

A typical protocol of filter-aided sample preparation (FASP) for tryptic digestion was carried out overnight at 37 °C using trypsin (Promega, Madison, WI, USA) both for whole tears and FACS-sorted EVs. Digested proteins from each sample were analyzed in triplicate by LC-MS/MS using a Proxeon EASY-nLCII (Thermo Fisher Scientific, Milan, Italy) chromatographic system coupled to a Maxis HD UHR-TOF (Bruker Daltonics GmbH, Bremen, Germany) mass spectrometer. Digested peptides were analyzed on the EASY-Column C18 trapping column (2 cm L., 100 µm I.D, 5 µm ps, Thermo Fisher Scientific, Milan, Italy), and subsequently on an Acclaim PepMap100 C18 (75 µm I.D., 25 cm L, 5 µm ps, Thermo Fisher Scientific, Milan, Italy) The flow rate was set to 0.300 µL/min with a total run time of 90 min. Chromatographic parameters and mass spectrometer setting conditions have been reported in our previous work [14].

### 4.8. Data Processing of Label Free Proteomics Analysis

PeptideShaker search engine platform was employed for the first interpretation of proteomics identification results using “X!Tandem” algorithm. Moreover, quantitative data analysis of FACS-sorted EVs was performed by a free computational proteomics platform, MaxQuant version 1.6.3.4. (Max-Planck Institute for Biochemistry, Martinsried, Germany), using raw data file of MS/MS spectra. Peak lists, generated in MaxQuant, were searched using Andromeda [39] peptide search engine against the UniProt database (released 2018_04, taxonomy *Homo Sapiens*, 20874 entries) supplemented with frequently observed contaminants and containing forward and reverse sequences. Multiplicity was set to one because a label free quantification was performed. Trypsin digestion mode was specified with up to two missed cleavages. Carbamidomethylation of cysteines (C) was defined as fixed modification and used in protein quantification, while oxidation of methionines (M) was set as variable modification. Minimum peptide length of 7 amino acids was set and the search space was limited to a maximum peptide mass of 4600 Da. MaxQuant uses individual mass tolerances for each peptide; the initial maximum precursor mass tolerances were set by default to 0.07 Da in the first search and 0.006 Da in the main search, and the fragment mass tolerance was set to 0.1 Da. A retention time tolerance of 2 min was used to align any time shift in acquisition between samples. False discovery rate (FDR) at the protein level was set at 3%, on the contrary at peptide level was set at 1%. Protein identification was performed with at least one unique peptide. Intensity-based absolute quantification (iBAQ) in MaxQuant was performed on the identified peptides to quantify protein abundance in the mixture.

Functional enrichment analysis of all identified proteins was obtained through Functional Enrichment analysis tool (FunRich software) in order to delineate specific Cellular Components involving the proteins identified in the tears of patients with POAG.

Differentially expressed EV proteins were further uploaded for “Core Analysis” through Ingenuity Pathway Analysis system (IPA, Qiagen, Hilden, Germany) to map statistically significative proteins for their functional annotation, as well as canonical pathway analysis, network discovery, upstream regulator analysis and downstream effects networks. We considered molecules and/or relationships in all species and a confidence setting as high predicted or experimental observed (excluding medium predicted). IPA quickly identifies relationships and pathways relevant to the uploaded dataset, providing disease and function categories based on the modulated proteins of the dataset. Moreover, upstream regulator analysis highlights the expected effects between transcriptional regulators and their target genes, as suggested by scientific literature in the IPA program [40]. The predicted activation or inhibition of each transcriptional regulators is inferred by z score generated by IPA system (z scores > 2.0 indicate that a molecule is activated, whereas z scores < −2.0 indicate the inhibition of target molecules).

### 4.9. Data Processing and Statistics

Metabolomics data were processed using NeoLynx software (Waters, Manchester, UK), and levels of AAs, ACCs, C0, succinyl acetone, nucleosides, and lysophospholipids were used to build a Orthogonal Partial Least Squares - Discriminant Analysis (orthoPLS-DA), using Metaboanalyst 3.0 software. The D’Agostino and Pearson omnibus normality test was performed for each metabolite. If normality test passed, Student’s t-test was used, otherwise Mann Whitney U-test was performed for comparisons between clinical groups by GraphPad Prism (GraphPad software, Inc, La Jolla, CA 92037, USA). Bean plots were obtained by the free online BoxPlotR software (http://shiny.chemgrid.org/boxplotr). The values of *p* < 0.05 were considered significant. The 95% of confidence interval was assumed for each test.

## Figures and Tables

**Table 1:** Demographics and clinical of naïve to therapy glaucomatous patients and control (CTRL).

Groups	Age (years ± SD)	Gender (M/F)	IOP (mmHg ± SD)	MD (dB ± SD)
POAG	64.63 ± 9.23	7/9	23 ± 1.55 *	−1.48 ± 2.80
CTRL	61.53 ± 8.53	7/10	19.41 ± 3.86	0.023 ± 2.11

IOP = intraocular pressure, MD = mean defect, POAG = primary open angle glaucoma, dB = decibel, M = male, F = female, SD = standard deviation. * *p* < 0.01 versus Controls.

## Supplementary Materials

The following are available online at https://www.mdpi.com/1422-0067/20/16/4029/s1.
