# Supplementary material for: Multi-Omics Approach for Studying Tears in Treatment-Naïve Glaucoma Patients
Source: Int J Mol Sci. 2019 Aug 18;20(16):4029. doi: 10.3390/ijms20164029 (PMC6721157; doi:10.3390/ijms20164029)
Supplement: Supplementary file 1 [file ijms-20-04029-s001.zip › ijms-535743-supplementary final_PROOF/Supplementary Table S1-S2-S5.docx]

Multi-Omics Approach for Studying Tears in Treatment-Naïve Glaucoma Patients

Claudia Rossi^1,2,^, Ilaria Cicalini^1,3^, Maria Concetta Cufaro^1,4^, Luca Agnifili^3,5^, Leonardo Mastropasqua^3,5^, Paola Lanuti^3^, Marco Marchisio^3^ Vincenzo De Laurenzi^1,2^, Piero Del Boccio^1,4^ and Damiana Pieragostino^1, 2^

^1^ Center for Advanced Studies and Technology (CAST), University ‘‘G. d’Annunzio’’ of Chieti-Pescara, Chieti, Italy.

^2^Department of Medical, Oral and Biotechnological Sciences, University ‘‘G. d’Annunzio’’ of Chieti-Pescara, Chieti, Italy.

^3^Department of Medicine and Aging Science, "G. d'Annunzio" University of Chieti-Pescara, Italy

^4^Department of Pharmacy, University ‘‘G. d’Annunzio’’ of Chieti-Pescara, Chieti, Italy.

^5^Opthalmic Clinic, Ss Annunziata Hospital 66100 Chieti, Italy.

*Correspondence to: Damiana Pieragostino, PhD, Departmentof Medical, Oral and Biotechnological Sciences, University ‘‘G. d’Annunzio’’ of Chieti-Pescara, Chieti, Italy.

[e-mail: damiana.pieragostino@unich.it](mailto:e-mail:%20damiana.pieragostino@unich.it)Phone: +39 0871 541593,Fax: +39 0871 541598

***Keywords:***Proteomics, Extracellular Vesicles, Metabolomics; Lipidomics; Tears; Biomarkers;AminoAcids; Acylcarnitines; Glaucoma

**Table S1.**List of media and standard deviation (SD) of tears amino acids (AA), free carnitine (C0), acylcarnitines (ACC), succinylacetone (SA), nucleosides and lysophospholipidsanalyzed. The table lists the p-value for each analyte in the comparison between glaucoma patients and healthy controls, the significant p-values are indicated in bold

|  | Glaucoma patients | | Healthy controls | |  |
| --- | --- | --- | --- | --- | --- |
| Analyte | Mean | SD | Mean | SD | P value |
| ALA | 191.84 | 62.63 | 255.96 | 70.50 | **0.03** |
| ARG | 55.31 | 18.91 | 86.16 | 34.12 | **0.01** |
| CIT | 28.42 | 17.24 | 34.99 | 35.20 | 0.56 |
| GLN\LYS | 374.29 | 105.95 | 537.45 | 163.50 | **0.007** |
| GLU | 244.92 | 64.20 | 313.23 | 104.34 | 0.06 |
| GLY | 214.94 | 70.33 | 280.40 | 115.41 | 0.10 |
| LEU\ILE\PRO-OH | 57.37 | 20.03 | 88.93 | 32.17 | **0.007** |
| MET | 18.94 | 5.94 | 25.71 | 6.87 | **0.01** |
| ORN | 43.47 | 19.47 | 57.59 | 35.38 | 0.23 |
| PHE | 22.50 | 10.61 | 35.02 | 14.52 | **0.02** |
| PRO | 77.08 | 27.01 | 109.15 | 34.43 | **0.01** |
| SA | 0.67 | 0.16 | 0.67 | 0.14 | 0.95 |
| TYR | 18.13 | 5.33 | 24.49 | 9.50 | 0.05 |
| VAL | 61.88 | 17.70 | 88.46 | 26.41 | **0.007** |
| ASA-Total | 1.18 | 0.31 | 1.17 | 0.41 | 0.96 |
| ADO | 7.51 | 8.90 | 5.74 | 5.66 | 0.54 |
| C0 | 8.77 | 3.44 | 11.90 | 4.27 | 0.05 |
| C10 | 0.07 | 0.08 | 0.05 | 0.01 | 0.59 |
| C10:1 | 0.03 | 0.01 | 0.03 | 0.01 | 0.64 |
| C10:2 | 0.03 | 0.005 | 0.03 | 0.01 | 0.17 |
| C2 | 5.05 | 1.70 | 7.34 | 2.38 | **0.008** |
| C3 | 1.46 | 0.64 | 1.80 | 0.88 | 0.28 |
| C3DC\C4OH | 0.09 | 0.04 | 0.11 | 0.03 | 0.24 |
| C4 | 0.23 | 0.09 | 0.26 | 0.07 | 0.42 |
| C4DC\C5OH | 0.07 | 0.02 | 0.07 | 0.02 | 0.30 |
| C5 | 0.60 | 0.49 | 0.82 | 0.60 | 0.33 |
| C5:1 | 0.05 | 0.04 | 0.04 | 0.01 | 0.46 |
| C5DC\C6OH | 0.14 | 0.08 | 0.24 | 0.16 | 0.09 |
| C6 | 0.04 | 0.02 | 0.04 | 0.01 | 0.27 |
| C6DC | 0.13 | 0.02 | 0.12 | 0.04 | 0.91 |
| C8 | 0.14 | 0.30 | 0.07 | 0.02 | 0.40 |
| C8:1 | 0.04 | 0.01 | 0.03 | 0.02 | 0.78 |
| D-ADO | 0.06 | 0.03 | 0.06 | 0.03 | 0.92 |
| C12 | 0.03 | 0.01 | 0.04 | 0.01 | 0.36 |
| C12:1 | 0.02 | 0.01 | 0.02 | 0.004 | 0.28 |
| C14 | 0.02 | 0.01 | 0.02 | 0.01 | 0.79 |
| C14:1 | 0.07 | 0.02 | 0.07 | 0.02 | 0.98 |
| C14:2 | 0.02 | 0.005 | 0.02 | 0.005 | 0.57 |
| C14OH | 0.01 | 0.005 | 0.01 | 0.005 | 0.80 |
| C16 | 0.03 | 0.01 | 0.03 | 0.01 | 0.17 |
| C16:1 | 0.01 | 1.8E-18 | 0.01 | 0.004 | 0.10 |
| C16:1OH\C17 | 0.03 | 0.01 | 0.03 | 0.01 | 0.89 |
| C16OH | 0.01 | 0.01 | 0.01 | 0.01 | 0.61 |
| C18 | 0.02 | 0.01 | 0.02 | 0.01 | 0.33 |
| C18:1 | 0.01 | 0.00 | 0.01 | 0.004 | 0.38 |
| C18:1OH | 0.03 | 0.01 | 0.03 | 0.01 | 0.64 |
| C18:2 | 0.01 | 0.003 | 0.01 | 0.00 | 0.21 |
| C18:2OH | 0.01 | 0.003 | 0.01 | 0.01 | 0.05 |
| C18OH | 0.01 | 0.003 | 0.01 | 0.003 | 0.91 |
| C20 | 0.01 | 0.01 | 0.01 | 0.005 | 0.89 |
| C20:0-LPC | 0.04 | 0.04 | 0.07 | 0.06 | 0.09 |
| C22 | 0.01 | 0.01 | 0.01 | 0.00 | 0.92 |
| C22:0-LPC | 0.04 | 0.03 | 0.08 | 0.07 | **0.02** |
| C24 | 0.02 | 0.01 | 0.01 | 0.00 | 0.30 |
| C24:0-LPC | 0.03 | 0.02 | 0.06 | 0.04 | **0.03** |
| C26 | 0.01 | 0.01 | 0.02 | 0.01 | 0.59 |
| C26:0-LPC | 0.02 | 0.01 | 0.03 | 0.03 | 0.38 |

**Table S2.** List of media and standard deviation (SD) of tear-gas amino acids (AA), free carnitine (C0), acylcarnitines (ACC), succinylacetone (SA), nucleosides and lysophospholipidsanalyzed. The table lists the p-value for each analyte in the comparison between female and male glaucoma patients.

|  | Glaucoma female patients | | Glaucoma male patients | |  |
| --- | --- | --- | --- | --- | --- |
| Analyte | Mean | SD | Mean | SD | P value |
| ALA | 236.310 | 143.962 | 248.850 | 155.059 | 0.883 |
| ARG | 72.451 | 45.951 | 74.267 | 57.651 | 0.951 |
| CIT | 27.169 | 20.199 | 84.727 | 121.270 | 0.239 |
| GLN\LYS | 495.309 | 345.123 | 409.663 | 176.176 | 0.595 |
| GLU | 302.451 | 129.610 | 256.325 | 109.226 | 0.507 |
| GLY | 236.817 | 102.017 | 320.488 | 255.140 | 0.440 |
| LEU\ILE\PRO-OH | 91.494 | 96.269 | 74.005 | 48.454 | 0.696 |
| MET | 29.794 | 29.622 | 17.257 | 7.726 | 0.338 |
| ORN | 42.143 | 10.054 | 71.620 | 55.892 | 0.195 |
| PHE | 32.556 | 29.397 | 26.208 | 14.441 | 0.641 |
| PRO | 110.087 | 108.350 | 92.345 | 43.444 | 0.715 |
| SA | 0.639 | 0.090 | 0.595 | 0.236 | 0.659 |
| TYR | 24.967 | 20.876 | 26.293 | 20.039 | 0.910 |
| VAL | 90.907 | 87.095 | 85.995 | 59.873 | 0.910 |
| ASA-Total | 1.259 | 0.324 | 1.075 | 0.300 | 0.315 |
| ADO | 6.631 | 7.917 | 9.360 | 10.333 | 0.600 |
| C0 | 14.336 | 15.148 | 8.852 | 2.963 | 0.404 |
| C10 | 0.049 | 0.016 | 0.088 | 0.119 | 0.397 |
| C10:1 | 0.034 | 0.008 | 0.033 | 0.008 | 0.835 |
| C10:2 | 0.033 | 0.005 | 0.030 | 0.006 | 0.377 |
| C2 | 6.990 | 4.864 | 5.320 | 2.487 | 0.465 |
| C3 | 2.181 | 1.533 | 1.383 | 0.693 | 0.266 |
| C3DC\C4OH | 0.116 | 0.047 | 0.090 | 0.054 | 0.377 |
| C4 | 0.296 | 0.151 | 0.232 | 0.051 | 0.344 |
| C4DC\C5OH | 0.086 | 0.047 | 0.065 | 0.027 | 0.363 |
| C5 | 0.920 | 0.651 | 0.525 | 0.314 | 0.203 |
| C5:1 | 0.047 | 0.015 | 0.057 | 0.061 | 0.695 |
| C5DC\C6OH | 0.193 | 0.156 | 0.198 | 0.133 | 0.947 |
| C6 | 0.044 | 0.017 | 0.048 | 0.021 | 0.712 |
| C6DC | 0.141 | 0.020 | 0.113 | 0.027 | 0.057 |
| C8 | 0.059 | 0.029 | 0.222 | 0.431 | 0.336 |
| C8:1 | 0.039 | 0.015 | 0.032 | 0.010 | 0.349 |
| D-ADO | 0.049 | 0.029 | 0.090 | 0.093 | 0.284 |
| C12 | 0.037 | 0.008 | 0.037 | 0.020 | 0.954 |
| C12:1 | 0.019 | 0.007 | 0.015 | 0.005 | 0.330 |
| C14 | 0.023 | 0.008 | 0.022 | 0.010 | 0.810 |
| C14:1 | 0.070 | 0.006 | 0.065 | 0.033 | 0.697 |
| C14:2 | 0.019 | 0.004 | 0.015 | 0.005 | 0.193 |
| C14OH | 0.007 | 0.005 | 0.007 | 0.005 | 0.867 |
| C16 | 0.034 | 0.029 | 0.040 | 0.018 | 0.687 |
| C16:1 | 0.014 | 0.011 | 0.012 | 0.004 | 0.604 |
| C16:1OH\C17 | 0.030 | 0.019 | 0.030 | 0.013 | 1.000 |
| C16OH | 0.014 | 0.008 | 0.013 | 0.005 | 0.805 |
| C18 | 0.020 | 0.018 | 0.030 | 0.018 | 0.342 |
| C18:1 | 0.014 | 0.011 | 0.015 | 0.008 | 0.901 |
| C18:1OH | 0.033 | 0.015 | 0.033 | 0.014 | 0.954 |
| C18:2 | 0.010 | 0.000 | 0.008 | 0.004 | 0.300 |
| C18:2OH | 0.010 | 0.000 | 0.008 | 0.004 | 0.300 |
| C18OH | 0.009 | 0.004 | 0.010 | 0.000 | 0.377 |
| C20 | 0.011 | 0.009 | 0.030 | 0.031 | 0.156 |
| C20:0-LPC | 0.043 | 0.056 | 0.045 | 0.048 | 0.943 |
| C22 | 0.007 | 0.005 | 0.015 | 0.012 | 0.145 |
| C22:0-LPC | 0.036 | 0.035 | 0.118 | 0.186 | 0.270 |
| C24 | 0.014 | 0.005 | 0.023 | 0.018 | 0.218 |
| C24:0-LPC | 0.050 | 0.067 | 0.085 | 0.141 | 0.569 |
| C26 | 0.013 | 0.005 | 0.018 | 0.012 | 0.280 |
| C26:0-LPC | 0.020 | 0.019 | 0.035 | 0.044 | 0.427 |

are available as supplementary files in excel format.

Table S3: Identified protein in whole biofluid from POAG patients and CTRL

Table S4: Identified protein in purified EVs from tears of POAG patients and CTRL

**Table S5.** Electrospray ionization mass spectrometry (ESI-MS) acquisition parameters employed for the analysis of tears amino acids (AAs), free carnitine (C0),acylcarnitines (ACCs), succinylacetone(SA), nucleosides and lysophospholipids. MS/MS transitions for each analysed metabolites and the corresponding internal standard (IS, shown in bold), the optimal cone potential (V), and collision energy (eV) are shown for each analyte

| **Abbreviation Amino acids**  **and Internal Standards** | **Amino acids** | **Transition** | **Cone potential** | **Collision energy** |
| --- | --- | --- | --- | --- |
| Ala  **^2^H_3_-Ala** | Alanine | 90.1>44.0  93.1>47.1 | 22 | 8 |
| Arg  **^2^H_4_, ^13^C-Arg** | Arginine | 175.1>70.1  180.1>75.1 | 34 | 21 |
| Asa  **^2^H_4_, ^13^C-Arg** | Argininosuccinic acid | 291.1>70.1  180.1>75.1 | 42  34 | 30  21 |
| Cit  **2H2-Cit** | Citrulline | 176.1>113.1  178.1>115.1 | 24 | 16 |
| Gly  **^15^N,2-^13^C-Gly** | Glycine | 76.0>30.0  78.0>32.0 | 22 | 7 |
| Leu/Ile/Pro-OH  **^2^H_3_-Leu** | Leucine/Isoleucine/Hydroxyproline | 132.1>86.1  135.1>89.1 | 24 | 10 |
| Met  **^2^H_3_-Met** | Methionine | 150.1>104.1  153.1>107.1 | 24 | 10 |
| Orn  **^2^H_6_-Orn** | Ornithine | 133.1>70.1  139.1>76.1 | 22 | 16 |
| Phe  **^13^C_6_-Phe** | Phenylalanine | 166.1>120.1  172.1>126.1 | 25 | 12 |
| Tyr  **^13^C_6_-Tyr** | Tyrosine | 182.1>136.1  188.1>142.1 | 26 | 12 |
| Val  **^15^N,2-^13^C_5_-Val** | Valine | 118.1>72.1  124.1>77.1 | 23 | 10 |
| **Pro**  **^13^C_5_-Pro** | Proline | 116.1>70.1  121.1>74.1 | 28 | 12 |
| **Gln/Lys**  **^13^C_5_-Gln** | Glutamine/Lysine | 147.1>84.0  152.1>88.1 | 22 | 16 |
| **Glu**  **^13^C_5_-Gln** | Glutamic acid | 148.1>84.0  152.1>88.1 | 24  22 | 14  16 |
| **Abbreviation Acylcarnitines**  **and Internal Standards** | **Acylcarnitines** | **Transition** | **Cone potential** | **Collision energy** |
| C0 | Free Carnitine | 162.1>1030 | 38 | 16 |
| **^2^H_9_-C0** |  | 171.2>103.0 |  |  |
| C2 | Acetylcarnitine | 204.1>85.0 | 34 | 18 |
| **^2^H_3_-C0** |  | 207.1>85.0 |  |  |
| C3 | Propionylcarnitine | 218.1>85.0 | 32 | 18 |
| **^2^H_3_-C3** |  | 221.2>85.0 |  |  |
| C4  C3DC/C4OH  **^2^H_3_-C4** | Butyrylcarnitine  Malonylcarnitine/3-Hydroxy-butyrylcarnitine | 232.2>85.0 248.1>85.0  235.2>85.0 | 36 | 18 |
| C5  C5:1  C4DC/C5OH  **^2^H_9_-C5** | Valerylcarnitine  Tiglylcarnitine  Methylmalonylcarnitine/3-Hydroxy-valerylcarnitine | 246.2>85.0 244.2>85.0 262.1>85.0  255.2>85.0 | 38 | 20 |
| C6  **^2^H_3_-C6** | Hexanoylcarnitine | 260.2>85.0 263.2>85.0 | 37 | 20 |
| C5DC/C6OH  C6DC  **^2^H_6_-C5DC** | Glutarylcarnitine/3-Hydroxy-hexanoylcarnitine  Adipylcarnitine | 276.2>85.0  290.2>85.0  282.2>85.0 | 40 | 24 |
| C8:1  C8  **^2^H_3_-C8** | Octenoylcarnitine  Octanoylcarnitine | 286.2>85.0  288.2>85.0  291.2>85.0 | 42 | 22 |
| C10 C10:2 C10:1  **^2^H_3_-C10** | Decanoylcarnitine  Decadienoylcarnitine  Decenoylcarnitine | 316.2>85.0  312.2>85.0  314.2>85.0  319.3>85.0 | 45 | 22 |
| C12  C12:1  **^2^H_3_-C12** | Dodecenoylcarnitine  Dodecanoylcarnitine | 344.3>85.0  342.3>85.0  347.3>85.0 | 46 | 24 |
| C14C14:1  C14:2  C14OH  **^2^H_3_-C14** | Tetradecanoylcarnitine (myristoylcarnitine)  Tetradecenoylcarnitine  Tetradecadienoylcarnitine  3-Hydroxy-tetradecanoylcarnitine | 372.3>85.0  370.3>85.0  368.3>85.0  388.3>85.0  375.3>85.0 | 52 | 25 |
| C16:1  C16  C16OH  **^2^H_3_-C16** | Hexadecenoylcarnitine  Hexadecanoylcarnitine (Palmitoylcarnitine)  3-Hydroxy-hexadecanoylcarnitine | 398.3>85.0  400.3>85.0  416.3>85.0  403.4>85.0 | 55 | 26 |
| C16:1OH/C17  **^2^H_3_-C16** | 3-Hydroxy-hexadecenoylcarnitine | 414.3>85.0  403.4>85.0 | 55 | 26 |
| C18  C18:1  C18:2  C18:1OH  C18OH  C18:2OH  **^2^H_3_-C18** | Octadecanoylcarnitine (Stearoylcarnitine)  Octadecenoylcarnitine (Oleylcarnitine)  Octadecadienoylcarnitine (Linoleylcarnitine)  3-Hydroxy-octadecenoylcarnitine  3-Hydroxy-octadecanoylcarnitine3-Hydroxy-octadecadienoylcarnitine | 428.4>85.0  426.4>85.0  424.3>85.0  442.4>85.0  444.4>85.0  440.3>85.0  431.4>85.2 | 56 | 28 |
| C20  **^2^H_3_-C26** | Eicosanoylcarnitine (Arachidoylcarnitine) | 456.4>85.0  543.5>85.0 | 69 | 34 |
| C22  **^2^H_3_-C26** | Docosanoylcarnitine (Behenoylcarnitine) | 484.4>85.0  543.5>85.0 | 69 | 34 |
| C24  **^2^H_3_-C26** | Tetracosanoylcarnitine (Lignoceroylcarnitine) | 512.5>85.0  543.5>85.0 | 69 | 34 |
| C26  **^2^H_3_-C26** | Hexacosanoylcarnitine (Cerotoylcarnitine) | 540.5>85.0  543.5>85.0 | 69 | 34 |
| **Abbreviation Ketones**  **and Internal Standards** | **Ketones** | **Transition** | **Cone potential** | **Collision energy** |
| SA  **^13^C_5_-MPP^2^** | Succinylacetone | 155.1>109.1  160.1>114.1 | 24 | 22 |
| **Abbreviation Nucleosides**  **and Internal Standards** | **Nucleosides** | **Transition** | **Cone potential** | **Collision energy** |
| ADO  **^13^C_5_-ADO** | Adenosine | 268.1>136.1  273.1>136.1 | 32 | 18 |
| D-ADO  **^13^C_5_-dADO** | 2’-deoxyadenosine | 252.1>136.1  257.1>136.1 | 29 | 14 |
| **AbbreviationLysophospholipidsand Internal Standards** | **Lysophospholipids** | **Transition** | **Cone potential** | **Collision energy** |
| C20:0-LPC  **^2^H_4_-C26:0-LPC** | C20:0 lysophosphatidylcholine | 552.4>104.1  640.5>104.1 | 74 | 30 |
| C22:0-LPC  **^2^H_4_-C26:0-LPC** | C22:0 lysophosphatidylcholine | 580.4>104.1  640.5>104.1 | 74 | 30 |
| C24:0-LPC  **^2^H_4_-C26:0-LPC** | C24:0 lysophosphatidylcholine | 608.5>104.1  640.5>104.1 | 74 | 30 |
| C26:0-LPC  **^2^H_4_-C26:0-LPC** | C26:0 lysophosphatidylcholine | 636.5>104.1  640.5>104.1 | 74 | 30 |
